# Abnormal Mitochondrial Function and Impaired Granulosa Cell Differentiation in Androgen Receptor Knockout Mice

**DOI:** 10.3390/ijms16059831

**Published:** 2015-04-30

**Authors:** Ruey-Sheng Wang, Heng-Yu Chang, Shu-Huei Kao, Cheng-Heng Kao, Yi-Chen Wu, Shuyuan Yeh, Chii-Reuy Tzeng, Chawnshang Chang

**Affiliations:** 1 Graduate Institute of Clinical Medicine & Department of Obstetrics and Gynecology, College of Medicine, Taipei Medical University, Taipei 110, Taiwan; E-Mails: d102094001@tmu.edu.tw (R.-S.W.); vvichern@yahoo.com.tw (Y.-C.W.); 2 Department of Biochemistry and Molecular Cell Biology, College of Medicine, Taipei Medical University, Taipei 110, Taiwan; E-Mail: hychang@tmu.edu.tw; 3 School of Medical Laboratory Science and Biotechnology, College of Medical Science and Technology, Taipei Medical University, Taipei 110, Taiwan; E-Mail: kaosh@tmu.edu.tw; 4 Center of General Education, Chang Gung University, Taoyuan 333, Taiwan; E-Mail: kao@mail.cgu.edu.tw; 5 George H. Whipple Lab for Cancer Research, Departments of Pathology, Urology and Radiation Oncology, University of Rochester Medical Center, Rochester, NY 14642, USA; E-Mail: shuyuan_yeh@urmc.rochester.edu

**Keywords:** androgen receptor, granulosa cell, PGC1-β, mitochondria

## Abstract

In the ovary, the paracrine interactions between the oocyte and surrounded granulosa cells are critical for optimal oocyte quality and embryonic development. Mice lacking the androgen receptor (*AR*^−/−^) were noted to have reduced fertility with abnormal ovarian function that might involve the promotion of preantral follicle growth and prevention of follicular atresia. However, the detailed mechanism of how AR in granulosa cells exerts its effects on oocyte quality is poorly understood. Comparing *in vitro* maturation rate of oocytes, we found oocytes collected from *AR*^−/−^ mice have a significantly poor maturating rate with 60% reached metaphase II and 30% remained in germinal vesicle breakdown stage, whereas 95% of wild-type *AR* (*AR^+/+^*) oocytes had reached metaphase II. Interestingly, we found these *AR*^−/−^ female mice also had an increased frequency of morphological alterations in the mitochondria of granulosa cells with reduced ATP generation (0.18 ± 0.02 *vs.* 0.29 ± 0.02 µM/mg protein; *p* < 0.05) and aberrant mitochondrial biogenesis. Mechanism dissection found loss of AR led to a significant decrease in the expression of peroxisome proliferator-activated receptor γ (PPARγ) co-activator 1-β (PGC1-β) and its sequential downstream genes, nuclear respiratory factor 1 (NRF1) and mitochondrial transcription factor A (TFAM), in controlling mitochondrial biogenesis. These results indicate that AR may contribute to maintain oocyte quality and fertility via controlling the signals of PGC1-β-mediated mitochondrial biogenesis in granulosa cells.

## 1. Introduction

Androgens classically mediate their genomic effects via the androgen receptor (AR), a protein encoded by an X chromosome gene, which exerts its biological function through activation of target gene expression via a sequence of processes [1,2]. The earlier studies indicated that androgens act directly on the development of ovarian follicles via AR, apart from serving as a substrate of aromatase (Cyp19a1) for estrogen synthesis. Androgens have been implicated to have a role in promoting follicular development [3,4], by up-regulating follicle-stimulating hormone (FSH) receptor (FSHR) expression and augmenting FSH-stimulated follicular differentiation [5,6,7,8]. In addition, AR is expressed in all cell types of the ovarian follicle, including granulosa cells, theca cells and the oocytes [9,10,11].

By using Cre/LoxP system, we and others generated the global *AR*^−/−^ [12,13,14] and granulosa cell-specific *AR*^−/−^ female mice [15,16]. Those data showed that female mice lacking AR have a reduced fertility, fewer oocytes were recovered after superovulation with gonadotropins, and fewer corpora lutea were observed. However, ablation of AR in oocytes had no effect on female fertility [15]. All together, these animal examples suggested that androgen, functions through the AR in granulosa cells, plays a primary and essential role for normal follicle development and optimal fertility. Earlier studies have shown that AR antagonists slow down mouse follicle growth [3,17] and prevent primary to secondary follicle transition in bovine [18]. Moreover, granulosa cell-specific *AR*^−/−^ female mice [15,16] have been found to contain more preantral follicles, more atretic follicles, with fewer antral follicles and corpora lutea in their ovaries. These animal examples suggest that androgen/AR signaling in granulosa cells regulates normal follicular growth, mainly by controlling preantral follicle growth and development to antral follicles, meanwhile preventing follicular atresia by inhibition of granulosa cell apoptosis. However, the definitive mechanisms of the subfertility and the potential pathways by which granulosa cell AR exerts its effects on follicle development and follicle atresia are still ill-defined.

Earlier studies suggested that mitochondria dysfunction in granulosa cells leads to poor oocyte quality and contribute to female subfertility [19,20,21]. In the current study, the prophase I arrested oocyte was collected from the ovary and the *in vitro* maturation rate of oocytes was evaluated as the preliminary indication for oocyte quality. In addition, oocyte maturation often required the energy sources from the surrounding nursing cells, such as granulose cells, as we reported here. Thus, the objective of this study was to determine whether lack of AR in granulosa cells has impacts on the oocyte quality by changing mitochondrial status and differentiation status in granulosa cells. We found alterations in mitochondrial morphology, biogenesis and metabolism in the granulosa cells of *AR*^−/−^ mice, suggesting that ablation of AR leads to mitochondrial dysfunction. In addition, the results in this study demonstrated that pregnant mare’s serum gonadotropin (PMSG)-induced granulosa cell differentiation was impaired in *AR*^−/−^ ovaries as indicated by decreasing the expression of genes involved in the granulosa cell differentiation. These consequences may be interrelated to significantly reduce *in* *vitro* maturation rate of *AR*^−/−^ oocytes.

## 2. Results

### 2.1. Confirmation of Knockout Androgen Receptor (AR) in the Ovaries in the AR^−/−^ Mice

**Figure 1:**
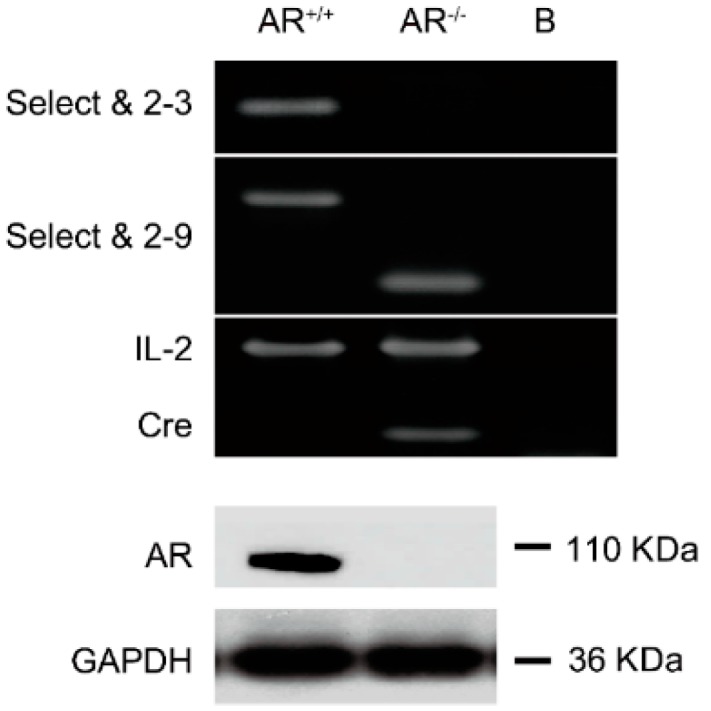
Genotyping of androgen receptor knockout (*AR*^−/−^) female mice. We used two pairs of primers (i) “select” and “2–3”; and (ii) “select” and “2–9” to identify *AR* wild type (*AR^+/+^*) and *AR* knockout (*AR*^−/−^) female mice in our study. Genotyping was performed by using PCR on the genomic DNA isolated from the tails of 3-week-old mice. “Select” is a forward primer which is located in the intron 1 of *AR* gene with sequence 5'-GTTGATACCTTAACCTCTGC-3'. “2–3” is a reverse primer which is located at the 3' end of the exon 2 with the sequence 5'-CTTCAGCGGCTCTTTTGAAG-3'. “2–9” is a reverse primer which is located in intron 2 with the sequence 5'-CTTACATGTACTGTGAGAGG-3'. Using the “select” and “2–3” primers, we amplified a product with ~460 bp for *AR^+/+^* allele, and with no product for *AR*^−/−^ allele. Using “select” and “2–9”, we amplified a DNA fragment with 580-bp, which represents *AR^+/+^* allele and ~270 bp, which represents *AR*^−/−^ allele (**upper panel**); The expression of Cre and internal control interleukin 2 (IL-2) were confirmed by PCR (bottom of upper panel). The lane marked B indicates blank control. Western blot shown that *AR*^−/−^ ovaries do not express AR protein (**lower panel**).

Three primers (“select”, “2–3” and “2–9”, for the relative position of each primer in the *AR* gene see Figure S1) were synthesized to amplify mouse genomic DNA to distinguish the floxed *AR*, *AR*^−/−^ and *AR^+/+^* mice. We were able to identify *AR*^−/−^ mice by using “select” and “2–9” primers to PCR amplify the 238-bp DNA (Figure 1, upper panel). In contrast, *AR^+/+^* mice can produce 580-bp DNA fragments by using “select” and “2–9” primers (Figure 1, upper panel). In this study, we were using mice with a ubiquitous deletion of the *AR*, so we further confirmed that *AR* was knocked out in ovaries by Western blot (Figure 1, lower panel).

### 2.2. Reduced Oocyte in Vitro Maturation Rate in AR^−/−^ Mice

**Figure 2:**
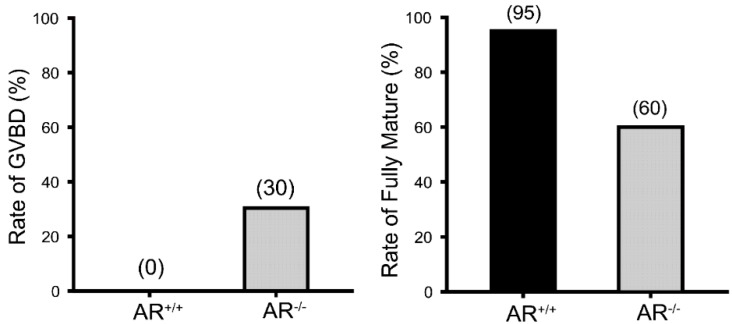
Reduced *in vitro* maturation rate of *AR*^−/−^ oocytes. Histogram shows the *in vitro* maturation rate of *AR^+/+^* and *AR*^−/−^ oocytes. After 18 h of *in vitro* culture, fewer *AR*^−/−^ oocytes could reach to metaphase II (60%) compared with *AR^+/+^* oocytes (95%) (**right panel**); In the meantime, about 30% of *AR*^−/−^ oocytes were remained in germinal vesicle breakdown (GVBD) stage (**left panel**), *n* = 100 follicles per genotype.

The *in vitro* oocyte maturation rate was examined to determine the potential AR roles in the oocyte maturation. The oocytes were collected from 4.5 weeks old *AR*^−/−^ and *AR^+/+^* female mice that were previously treated with PMSG for 48 h. The result revealed that 95% of *AR^+/+^* oocytes had reached to metaphase II, whereas a significantly lower maturation rate (60%) was observed in the *AR*^−/−^ oocytes (Figure 2, right panel). In the meantime, about 30% of *AR*^−/−^ oocytes were arrested at prophase I and remained at germinal vesicle breakdown (GVBD) stage (Figure 2, left panel).

### 2.3. Change of Ovarian Follicle Morphology in AR^−/−^ Mice

**Figure 3:**
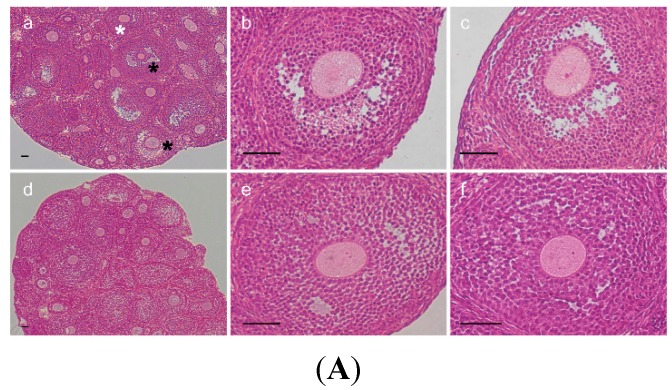

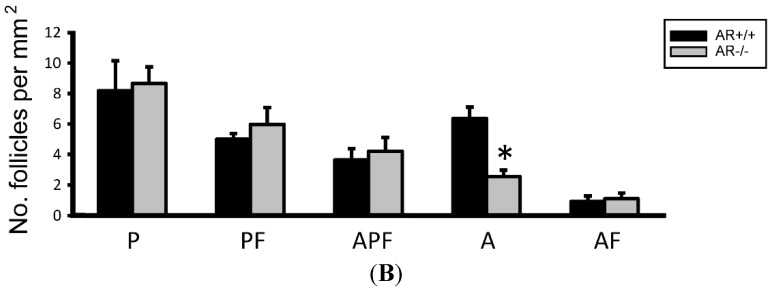
Morphological changes of ovarian follicle in *AR*^−/−^ mice. (**A**) Comparison of morphology in ovaries of 4.5-week-old *AR^+/+^* and *AR*^−/−^ female mice after PMSG-priming for 48 h (*n* = 4 mice per genotype). Representative hematoxylin and eosin-stained ovarian sections (**a**: *AR^+/+^*, **b**,**c**: *AR^+/+^*, **d**: *AR*^−/−^ at 100× magnification; **e**,**f**: *AR*^−/−^ at 400× magnification). The largest follicles had reached to Pedersen class 6 (incipient antral, white asterisk) or Pedersen class 7 (early antral, black asterisk) stages in *AR^+/+^* ovaries (**a**–**c**), whereas in *AR*^−/−^ ovaries, the majority of follicles did not show antrum formation in this time period (**d**–**f**). Scale bar, 50 μm; (**B**) Statistical analysis of the number of the follicular compartments in *AR^+/+^* and *AR*^−/−^ ovaries. The *AR*^−/−^ female mice have few antral follicles relative to *AR^+/+^* mice. P, Primordial and primary follicle; PF, Preantral follicle; APF, Atretic primordial, primary, and preantral follicle; A, Antral follicle; AF, Atretic antral follicle. * *p* < 0.05, by Student’s *t* test.

In order to elucidate whether poorer oocyte maturation rate in *AR*^−/−^ mice was linked with morphological change of ovarian follicles, the morphology of ovarian follicles were checked with HE staining. Figure 3A shows follicle morphology after superovulation treatment. The largest follicles had reached to Pedersen class 6 (incipient antral) or Pedersen class 7 (early antral) stages in *AR^+/+^* ovaries, whereas in *AR*^−/−^ ovaries, the majority of follicles did not show antrum formation in this time period. In agreement with our previous data [12], *AR*^−/−^ ovaries contained considerably few antral follicles as compared to *AR^+/+^* littermates (Figure 3B).

### 2.4. Alteration of Mitochondrial Morphology and Ultrastructure in Granulosa Cells from AR^−/−^ Mice

**Figure 4:**
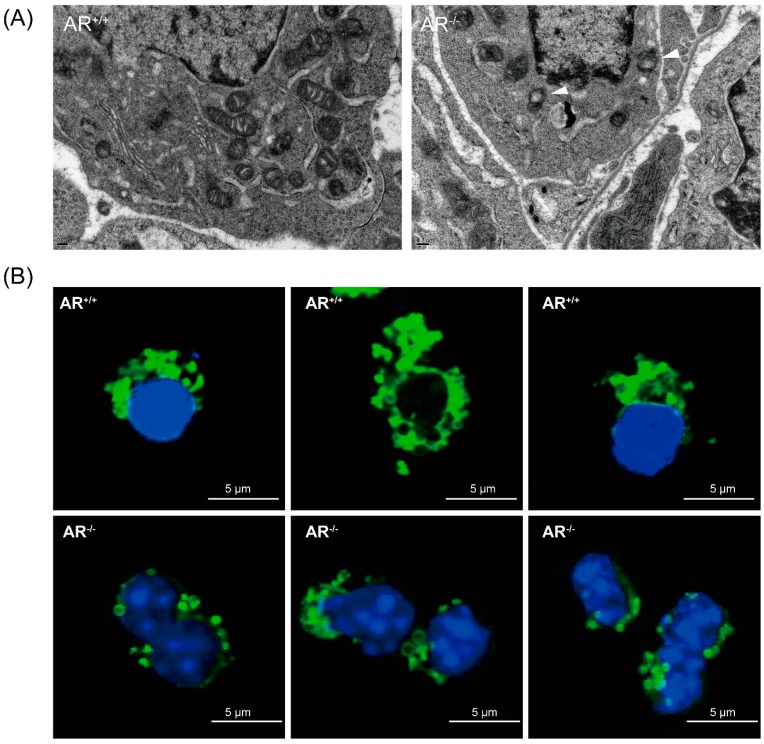
Alteration of mitochondrial morphology in granulosa cells of *AR*^−/−^ mice. (**A**) Mitochondrial morphology in granulosa cells of *AR*^−/−^ mice was visualized by transmission electron microscopy (TEM). Representative electron micrograph of mitochondria from control (*AR^+/+^*) granulosa cells, showing bean-shaped structures with numerous transversely orientated cristae enveloped by an intact outer membrane; *AR*^−/−^ granulosa cells displayed small spherical structures with fewer and disarrayed cristae, and the presence of large vacuoles in mitochondria (white arrowhead); (**B**) Mitochondrial morphology in granulosa cells of *AR*^−/−^ mice was visualized by immunofluorescence. Granulosa cells collected from *AR^+/+^* and *AR*^−/−^ mice were labeled with MitoTracker Green to visualize mitochondrial localization and co-stained with 4',6'-diamidino-2-phenylindole (DAPI) to visualize nuclei. Representative confocal sections of granulosa cells were shown. Mitochondria in granulosa cells from *AR*^−/−^ mice displayed aggregating distribution pattern and small spherical structures.

To reveal the molecular mechanisms of ovarian follicles morphology changes, and impact on oocyte quality, we assayed the damage on the mitochondria of granulosa cells since the mitochondria are important for steroidogenisis and energy production [22,23]. We first examined the mitochondrial ultrastructure in granulosa cells using TEM on the ovaries of both *AR^+/+^* and *AR*^−/−^ mice and found that most mitochondria of *AR^+/+^* granulosa cells contained highly folded inner membrane forming mitochondrial cristae, which were enveloped by an intact outer membrane. In contrast, the mitochondria of *AR*^−/−^ granulosa cells showed increase electron density of the matrix, generally more round in appearance with fewer, disarrayed cristae, and contained vacuoles (white arrowhead in Figure 4A). Immunofluorescent staining of mitochondria exhibited a normal perinuclear distribution in *AR^+/+^* granulosa cells. In contrast, the mitochondria in *AR*^−/−^ granulosa cells displayed aggregating distribution pattern and small “donut-like” vesicles appearance (Figure 4B).

**Figure 5:**
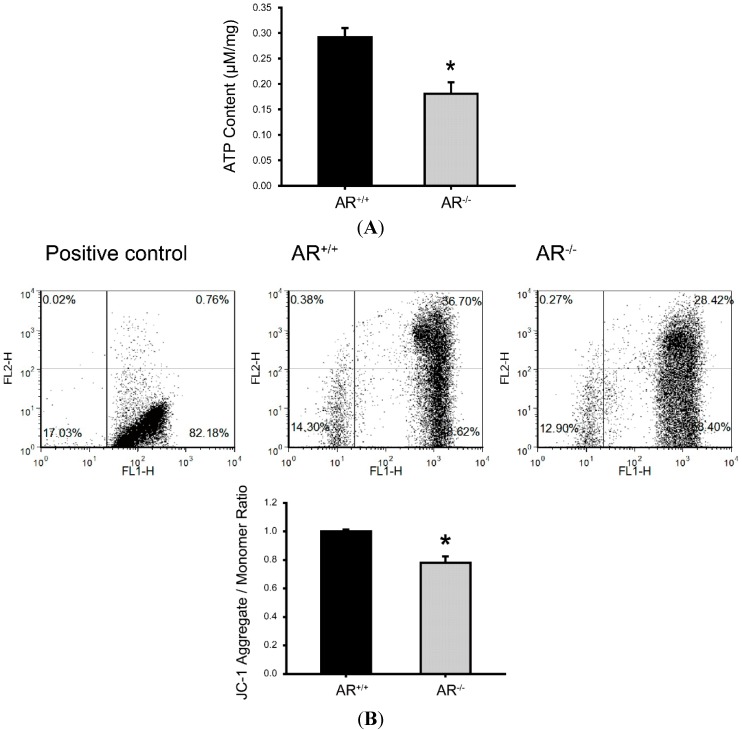
Metabolic dysfunction of mitochondria and reduced mitochondrial membrane potential in granulosa cells of *AR*^−/−^ mice. (**A**) Reduced ATP content in granulosa cells of *AR*^−/−^ mice. Granulosa cells removed from *AR^+/+^* and *AR*^−/−^ mice were collected to determine the levels of ATP. Values are expressed as µM per mg protein. Histogram shows the average ATP content in granulosa cells from *AR^+/+^* and *AR*^−/−^ mice. * *p* < 0.05, *n* = 3 mice per genotype; (**B**) Detection of mitochondrial membrane potential by flow cytometry analysis of JC-1 staining. Granulosa cells were collected from 4-week-old female mice (*AR^+/+^* and *AR*^−/−^) injected with 48 h of 7.5 IU pregnant mare’s serum gonadotropin (PMSG) treatment, and then stained with 5,5',6,6'-tetrachloro-1,1',3,3'-tetraethylbenzimidazolylcarbocyanine iodide (JC-1). JC-1 exists as a monomer in the cytosol (green fluorescent) and also accumulates as aggregates in the mitochondria (red fluorescent). In apoptotic and necrotic cells, JC-1 exists in monomeric form and stains the cytosol green. Results are expressed as the ratio of the aggregate to monomeric form of JC-1. The percentage is expressed as the mean ± SD (*n* = 3 mice per genotype). * *p* < 0.05.

### 2.5. Metabolic Dysfunction of Mitochondria and Reduced Mitochondrial Membrane Potential in Granulosa Cells of AR^−/−^ Mice

Based on the above findings, we determine whether mitochondrial metabolism and mitochondria membrane potential were disturbed in granulosa cells of *AR*^−/−^ mice. ATP synthesis by oxidative phosphorylation is the primary function associated with mitochondrial function [24]. Thus, the granulosa cells of *AR^+/+^* and *AR*^−/−^ mice were processed to measure ATP content. As shown in Figure 5A, the ATP content were markedly decreased in the granulosa cells of *AR*^−/−^ mice as compared to *AR^+/+^* mice (0.18 ± 0.02 µM/mg protein *vs.* 0.29 ± 0.02 µM/mg protein; *p* < 0.05). To detect the mitochondria membrane potential, we used flow cytometry analysis of JC-1 staining. JC-1 exists as a monomer in the cytosol (green fluorescent) and also accumulates as aggregates in the mitochondria (red fluorescent). In apoptotic and necrotic cells, JC-1 exists in monomeric form and stains the cytosol green. Results are expressed as the ratio of the aggregate to monomeric form of JC-1. As shown in Figure 5B, the mitochondria membrane potential were markedly reduced in the granulosa cells of *AR*^−/−^ mice as compared to *AR^+/+^* mice, which suggesting a decline of mitochondrial function.

### 2.6. Decreased Mitochondrial Biogenesis in Granulosa Cells of AR^−/−^ Mice

**Figure 6:**
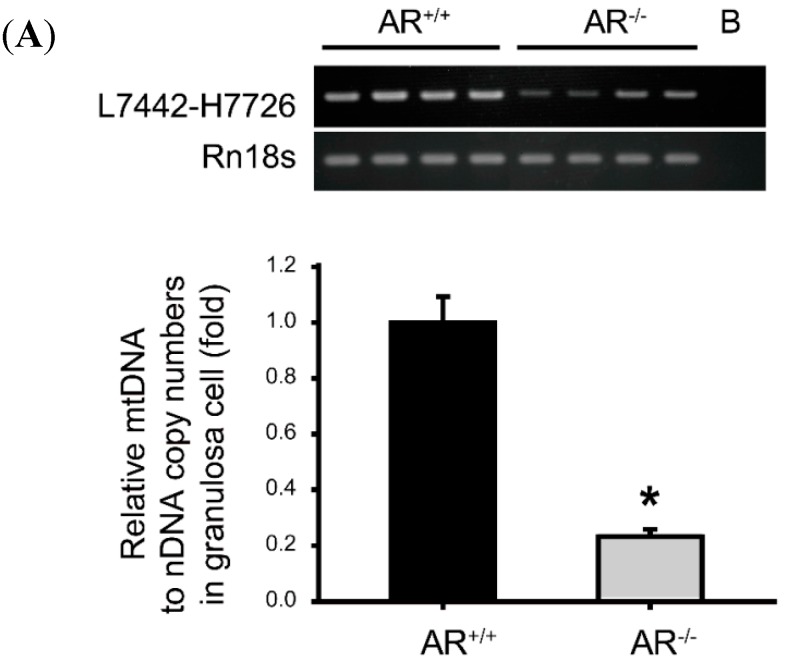

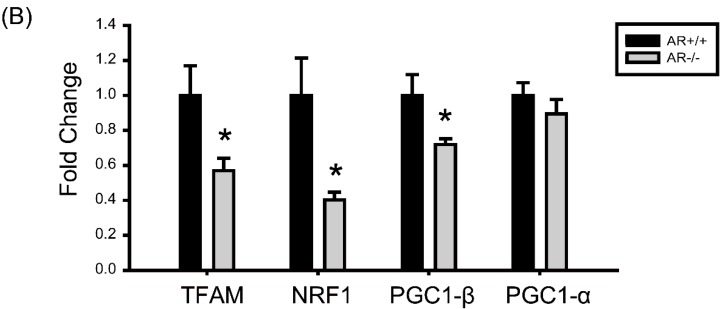
Decreased mitochondrial biogenesis in granulosa cells of *AR*^−/−^ mice. (**A**) Decreased mitochondrial DNA (mtDNA) copy number in granulosa cells of *AR*^−/−^ mice. mtDNA copy number was calculated using reverse transcription-PCR and quantitative real-time PCR by measuring the ratio of COX 2 (mtDNA) to Rn18s (nuclear DNA) DNA levels in granulosa cells of *AR^+/+^* and *AR*^−/−^ mice. * *p* < 0.05, *n* = 4 mice per genotype; the lane marked B indicates blank control; (**B**) Decreased mitochondrial biogenesis in granulosa cells of *AR*^−/−^ mice. The mRNA levels of genes implicated in mitochondrial biogenesis determined by real-time RT-PCR in granulosa cells of *AR^+/+^* and *AR*^−/−^ mice. At least three experiments were performed and data are presented as the mean ± SEM. * *p* < 0.05, *n* = 4 mice per genotype. PGC-1: peroxisome proliferator-activated receptor γ coactivator-1. NRF1: nuclear respiratory factor 1. TFAM: mitochondrial transcription factor A.

To investigate the potential effects of loss of AR in granulosa cells on mitochondrial biogenesis, we evaluated mtDNA content in granulosa cells from *AR^+/+^* and *AR*^−/−^ mice by quantitative real-time PCR. Data are expressed as the ratio of mtDNA to nuclear DNA, as shown inFigure 6A (lower panel). We found that mtDNA content in granulosa cells were significantly lower in *AR*^−/−^ mice than in *AR^+/+^* mice (0.2 ± 0.12 *vs.* 1.1 ± 0.2; *p* < 0.05). Figure 6A (upper panel) shows the mtDNA content by RT-PCR. Furthermore, we measured the mRNA levels of genes implicated in mitochondrial biogenesis, such as PGC-1α, PGC-1β, NRF1 and TFAM [25]. We found the granulosa cells of *AR*^−/−^ mice exhibited decreased expression of PGC-1β, TFAM and NRF1 mRNA compared with *AR^+/+^* mice (Figure 6B). No significant difference was detected for PGC-1α transcripts (Figure 6B). Together these results revealed a deterioration of mitochondrial biogenic response in granulosa cells of *AR*^−/−^ mice.

### 2.7. Molecular Changes in Granulosa Cells of AR^−/−^ Mice and the Effect of AR Deficiency on Serum E2 Levels

**Figure 7:**
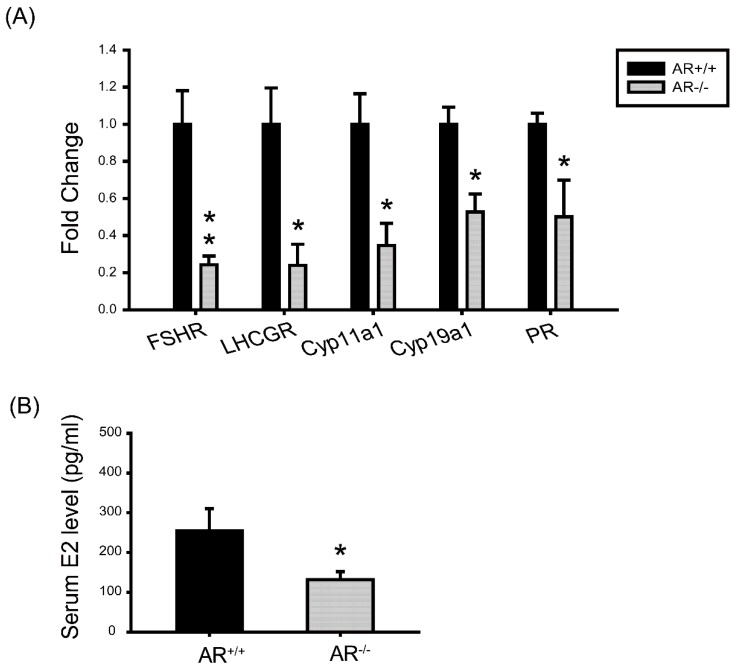
Molecular changes in granulosa cells of *AR*^−/−^ mice and the effect of AR deficiency on serum estradiol (E2) levels. (**A**) The mRNA levels of genes that are important in granulosa cell differentiation during folliculogenesis and luteinization. At least three experiments were performed and data are presented as the mean ± SEM (*n* = 4 mice per genotype) of the fold changes; (**B**) Serum E2 level was measured by ELISA. (*n* = 4 mice per genotype). Data was expressed as the mean ± SEM. * *p* < 0.05, ** *p* < 0.01. FSHR: FSH receptor; LHCGR: Luteinizing hormone receptor; Cyp11a1: Cytochrome P450 side-chain-cleavage; Cyp19a1: Cytochrome P450 aromatase; PR: Progesterone receptor.

To understand the defects in granulosa cells during gonadotropin stimulation, we examined expression of genes that were important granulosa cell proliferation and differentiation markers during folliculogenesis. Granulosa cell differentiation is manifested during the process allowing the progression of preantral follicle to preovulatory follicle, which is dependent on sufficient FSH stimulation [26,27,28] and is marked by the acquisition of increased Cyp19a1 activity and luteinizing hormone (LH) receptor (LHCGR). Meanwhile, since androgen is known to augment the actions of FSH [3,4,7], we are specifically interested in evaluating the expression pattern of the genes encoding FSHR, LHCGR, and Cyp19a1 in *AR^+/+^* and *AR^−/−^* ovaries. A number of genes showed significant changes. The FSHR, LHCGR, Cyp11a1, Cyp19a1 and progesterone receptor (PR) expression were significantly decreased in granulosa cells of *AR*^−/−^ mice (Figure 7A). These data indicate that preovulatory granulosa cells do not properly differentiate and develop sufficient aromatase activity in response to PMSG. Concomitantly, the level of serum estradiol (E2) was significantly decreased in *AR*^−/−^ female mice as compared with *AR^+/+^* littermates (Figure 7B).

## 3. Discussion

The primary cause of reduced fecundity in *AR*^−/−^ female mice is infrequent and inefficient ovulation [12,13,14,15,16]. In this study, we found a high frequency of morphological anomalies in the mitochondria of *AR*^−/−^ granulosa cells. Mitochondria displayed an increased electron density of the matrix, small spherical structures with fewer, disarrayed cristae, and containing vacuoles under TEM (Figure 4A). It also displayed an aggregated distribution and a small “donut-like” vesicles appearance under immunofluorescence staining (Figure 4B) in the granulosa cells of *AR*^−/−^ mice. These morphological changes are frequently referred to as “fragmented mitochondria” [29]. Evidences have suggested that fusion and fission of mitochondria affects the ability of cells to distribute their mitochondria to specific subcellular locations. It has been stated that trafficking of mitochondria are important for cellular function by placing them in appropriate locations relative to energy requiring processes [30]. Therefore, those fragmented and misplaced mitochondria in the granulosa cells of *AR*^−/−^ mice might result in sub-cellular energy deficiency and even cell death [31,32]. Mitochondrial biogenesis requires the symphonious expression of mtDNA and nuclear genes that encode both mitochondrial proteins and their regulatory factors [33]. It has been proved that the PGC-1 family regulates mitochondrial biogenesis by serving as a coactivator of multiple transcription factors, such as NFR1 and TFAM [34,35]. Our quantitative real-time PCR analysis revealed that mtDNA copy number was significantly decreased in granulosa cells from *AR*^−/−^ mice when compared to *AR^+/+^* mice. In agreement, PGC-1β, NRF1 and TFAM mRNAs were down-regulated in the *AR*^−/−^ granulosa cells (Figure 6B). In the mean time, the average ATP contents and mitochondrial membrane potential were markedly reduced in the granulosa cells of *AR*^−/−^ mice as compared to *AR^+/+^* mice (Figure 5A,B), suggesting a decline of mitochondrial function.

Within the follicle, cumulus cells surround the oocyte and directly contact with the oocyte via gap junctions [36]. The paracrine interactions between the oocyte and granulosa cells are bi-directional and critical for the development of granulosa cells, optimal oocyte quality and embryonic development [37,38]. Furthermore, earlier study has shown that oocytes enclosed by cumulus cells have higher ATP levels than those lacking cumulus cells [39], suggesting that cumulus cells provide ATP for oocyte development. Therefore, mitochondrial function is critical for the energy production in granulosa cells and then to participate as an energy source for oocyte maturation. The role of mitochondria in oocyte, in particular in the immature oocyte has not yet been identified. However, it is generally believed that mitochondria in the either oocyte or egg are in a transmitted state and undifferentiated. Therefore during oocyte maturation, the energy sources are believed from the surrounding cells [40]. Thus, in the current study, we demonstrated that the AR insufficiency that impact on mitochondria function will result in poor oocyte maturation rate. We found that 95% of *AR^+/+^* oocytes had reached to metaphase II, whereas a significantly lower maturation rate (60%) was observed in the *AR*^−/−^ oocytes. This is consistence with earlier studies that such a declined ATP content in oocytes has been suggested to significantly affect oocyte quality, embryonic development and even the implantation process [41,42]. We speculated that the decreased ATP levels and reduced mitochondria membrane potential in the granulosa cells of *AR*^−/−^ mice might contribute to the decreased oocyte competence to undergo final maturation, as observed in this study.

Our results also showed that PMSG-stimulated *AR*^−/−^ granulosa cells exhibit reduced FSHR, Cyp11a1 and Cyp19a1 mRNA expressions (Figure 7A). It has been reported, in the both of hypophysectomized rat [43], as well as mutant mice that lacking either gonadotrophins [44], FSH [26], or FSHR [27,28], causes follicular development arrest at the preantral stage. Androgens, via the activation of AR, can increase the expression of FSHR and augment FSH-stimulated follicular differentiation [5,6,7,8]. Cyp11a1 and Cyp19a1 are stimulated by FSH in granulosa cells as part of the differentiation program induced by this hormone [45]. Earlier study by Wu [46] nicely showed that androgens have a direct stimulatory effect on Cyp19a1 and Cyp11a1 expression in rat ovarian granulosa cells. In agreement with those earlier studies, our results provide the clear evidence that AR is required for maximum FSH stimulation of Cyp11a1 and Cyp19a1 activity. Our results also showed that the level of serum E2 was significantly decreased in *AR*^−/−^ mice as compared with *AR^+/+^* littermates (Figure 7B). Another important characteristic of preovulatory granulosa cells is the acquisition of LHCGR and PR, which provide the mechanism of follicle selection by which follicles can respond to the LH surge and ovulate [47]. Induction of LHCGR [48,49] and PR [50,51] expression in preovulatory granulosa cells is primarily activated by FSH. Herein, our data demonstrated that AR mediates this effect of androgen on FSHR expression further onwards regulation of LHCGR and PR levels in preovulatory granulosa cells. Granulosa cells of *AR*^−/−^ mice exhibit a reduced LHCGR and PR mRNA levels relative to *AR^+/+^* controls (Figure 7A). We proposed that the reduced LHCGR levels in the granulosa cells of *AR*^−/−^mice might contribute to compromised response to LH surge and oocyte competence to undergo final maturation.

Combining our previous findings [12] with the results presented here, we conclude that *AR*^−/−^ mice exhibit a reduced fertility with defective folliculogenesis and reduced corpus luteum formation might have been contributed by the following reasons. Mitochondrial dysfunction in granulosa cells may compromise the competence of oocytes in *AR*^−/−^ ovaries. These data indicate that the intraovarian actions of AR and androgen are critical for enhancing the FSH action and the generation of fully differentiated follicles that exhibit the proper cellular organization (*i.e.*, antrum formation and cumulus-oocyte complex), optimal mitochondrial function, the necessary enzymatic activity, and the essential receptor signaling pathways. All of those factors converge to provide the follicle with the normal capacity to respond to the LH surge and expel a healthy oocyte that is fully competent for fertilization.

## 4. Materials and Methods

All the culture media and chemicals were purchased from Sigma-Aldrich Chemical Company, unless otherwise stated.

### 4.1. Generation of Female AR^−/−^ Mice

All mouse studies were approved by the Animal Studies Committee at Taipei Medical University and conformed to the Guide for the Care and Use of Laboratory Animals published by the National Institutes of Health. *AR*^−/−^ female mice and heterozygous female mice (*AR*^+/−^) were generated by crossing AR-flox mice (exon 2 flanked by loxP sites) with transgenic β-actin-Cre (ACTB-Cre) mice, as previously described [52,53]. Briefly, male mice carrying floxed AR were mated with females genotyped with *AR/ar* ACTB-Cre (*AR*^+/−^) to produce female *AR*^−/−^ mice carrying the genotype *ar/ar* ACTB-Cre. Genotype of offspring was confirmed by PCR. Based on the sequence of the AR genomic DNA, three primers have been designed to distinguish the *AR^+/+^*, *AR*^−/−^, and floxed *AR* X chromosome on mice. The primer “select” is the 5' primer which is located in the intron 1 and its sequence is 5'-GTTGATACCTTAACCTCTGC-3'. The primer “2–9” is the 3' end primer which is located in intron 2 and its sequence is 5'-CTTACATGTACTGTGAGAGG-3'. The “2–3” primer is the 3' end primer which is located in the exon 2 and its sequence is 5'-CTTCAGCGGCTCTTTTGAAG-3'. If the mouse contains *AR^+/+^*, the primer pairs (select and “2–9”) will generate a product with 580 bp and the other pair of primers (select and “2–3”) will generate a product with ~460 bp. If the mice carry *AR*^−/−^, the primer pairs (select and “2–9”) will generate a product with ~270 bp and the other pair of primers (select and “2–3”) will not have a product. The expression of Cre and internal control interleukin 2 (IL-2) were confirmed by PCR during genotyping. The primer design and PCR conditions of Cre and IL-2 follow The Jackson Laboratory’s suggestions as previously described [52,53].

### 4.2. Western Blot Analysis

To determine the expression levels of AR, the protein extracts of ovaries were subjected to sodium dodecyl sulfatepolyacrylamide denaturing gel electrophoresis. Proteins were transferred to hybond-P PVDF membranes (GE Healthcare, Little Chalfont, UK) and processed by routine procedures. Immunoreactive bands were visualized by blotting with primary antibodies against AR (1:1000, LS-C137965, LifeSpan Biosciences, Seattle, WA, USA), followed by incubation with horseradish peroxidase-conjugated secondary antibodies and detection with enhanced chemiluminescence (Thermo Scientific, Rockford, IL, USA).

### 4.3. Tissue Sampling, Oocyte in Vitro Maturation Assay, RNA Extraction and Analysis

To collect fully grown germinal vesicle (GV) oocytes and granulosa cells, a minimum of six mice per genotype (4.5 weeks old; *AR*^−/−^ and *AR^+/+^*) were treated with 7.5 IU PMSG by intraperitoneal injection. After 48 h, the ovaries were removed and placed in a dish containing M2 medium. For the oocyte collection, cumulus-oocyte complexes (COCs) and granulosa cells were harvested by manually puncturing of large antral follicles with a sterile needle. Oocytes were denuded with gentle pipetting with pulled-glass Pasteur pipettes as previously described [54]. Oocytes were then cultured in M2 media on the 37 °C heated stage for another 18 h in order to score maturation rates. For collection of granulosa cells, ovarian debris was removed by filtered cells through a 40 µm nylon mesh filter. Granulosa cells were collected by centrifugation and processed for total RNA extraction using an RNeasy kit (Qiagen, Chatsworth, CA, USA). The final concentration of granulosa cells is approximately about 1.5 million per mL. Granulosa cells represent a mixture of both mural and cumulus cells and are referred to as “granulosa cells” in the text. One μg of total RNA was reverse transcribed and subjected to real-time PCR using LightCycler 2.0 instrument (Roche Applied Science, Mannheim, Germany). In general, the real-time PCR was performed with SYBR Green PCR Master Mix (Roche Applied Science). Each sample was run in triplicate. Data were analyzed by a LightCycler Software 4.0 (Roche Applied Science). The mRNA levels of genes of interest in *AR*^−/−^ mice were compared with those of their *AR^+/+^* littermates. Each gene expression pattern was confirmed using at least three pairs of *AR*^−/−^ and *AR^+/+^* mice. Primer sequences used for studying gene expression were designed by Beacon Designer II software (Bio-Rad Lab., Irvine, CA, USA) and are listed below: PPARγ co-activator 1-α (PGC-1α)-forward (f): GACATAGAGTGTGCTGCTCTGGT; PGC-1α-reverse (r): GTTCGCAGGCTCATTGTTGT; PGC-1β-f: CGCTCCAGGAGACTGAATCCAG; PGC-1β-r: CTTGACTACTGTCTGTGAGGC; NRF1-f: TTACTCTGCTGTGGCTGATGG; NRF1-r: CCTCTGATGCTTGCGTCGTCT; TFAM-f: AGTTCATACCTTCGATTTTC; TFAM-r: TGACTTGGAGTTAGCTGC; FSHR-f: GAACGCCATTGAACTGAGATT; FSHR-r: CGGAGACTGGGAAGATTCTG; LHCGR-f: AGTCCATCACGCTGAAACTGT; LHCGR-r: GGCCTGCAATTTGGTGGAAG; Cytochrome P450 side-chain-cleavage (Cyp11a1)-f: GGTGGACACGACCTCCATGA; Cyp11a1-r: TGCTGGCTTTGAGGAGTGGA; Cyp19a1-f: TTCGCTGAGAGACGTGGAGA; Cyp19a1-r: AGGATTGCTGCTTCGACCTC; Progesterone receptor (PR)-f: CTCATGAGTCGGCCAGAGAT; PR-r: CACTGTCCTCTTCCACCTCC; β2-microglobulin (β2m)-f: ACCCTCATCAATGGCCTGTGGA; β2m-r: CATGGGCTTTGACCCTTGGG. Mouse β2m was used as a housekeeping gene.

### 4.4. Histology

Ovaries from 4.5-week-old *AR*^−/−^ and *AR^+/+^* littermates (*n* = 4 per genotype) were obtained from PMSG treated animals. Ovaries were fixed in 4% paraformaldehyde at 4 °C and then paraffin embedded. Thereafter, 5-µm sections were taken at 30-µm intervals, mounted on slides, and subjected to hematoxylin and eosin staining for histological examination by light microscopy. All follicles with a visible oocyte nucleus were subsequently classified by stage of development. The follicle classification system was based on Pedersen’s system [55]: (1) Primordial follicles—identified by an oocyte surrounded by a layer of flattened granulose progenitor cells; (2) Pedersen class 3 (primary)—identified by an oocyte surrounded by a layer of cuboid granulosa cells. There are 21 to 60 granulosa cells on the largest cross-section; (3) Pedersen classes 4–5 (secondary)—identified by an oocyte surrounded by two or more layers of granulosa cells. There are 61 to 400 granulosa cells on the largest cross-section; (4) Pedersen class 6 (incipient antral)—identified by a large oocyte with many layers of granulosa cells. The granulosa cells are separated by scattered areas of fluid. There are 401 to 600 granulosa cells on the largest cross-section; (5) Pedersen class 7 (early antral)—a follicle with a single cavity containing follicle fluid. There are more than 600 cells on the largest cross-section. The cumulus oophorus has formed; (6) Pedersen class 8 (Graafian follicle)—a large follicle with a single cavity with follicle fluid and a well-defined cumulus stalk. There are more than 600 cells on the largest cross-section. Follicles were considered as atretic based on any of the two morphometric criteria within a single cross section: A degenerated oocyte, three or more pyknotic nuclei or atretic bodies in granulosa cell layers or follicular antrum, disorganized granulosa cell layers, granulosa cells pulling away from basement membrane, and broken basement membrane as previously described [15,56].

### 4.5. Transmission Electron Microscopy (TEM)

For ultrastructural analysis of mitochondria, the ovaries from *AR*^−/−^ and *AR^+/+^* mice were collected followed by 48 h PMSG treatment then fixed in a mixture of glutaraldehyde (1.5%) and paraformaldehyde (1.5%) in phosphate buffer at pH 7.3. They were then post-fixed in 1.0% osmium tetroxide, 1.5% potassium hexanoferrate, before being rinsed in cacodylate and 0.2 M sodium maleate buffers (pH 6.0) followed by block-staining with 1% uranyl acetate. Following dehydration, the ovaries were embedded in Epon and sectioned for TEM. Thin sections were cut, mounted on 200-mesh grids, stained with uranyl acetate and lead citrate, and examined using a H7100 Hitachi electron microscope (H7100, Hitachi High-Technologies Corporation, Tokyo, Japan). Digital images were captured using a MegaView III digital camera (OSIS Pro Software, Olympus Soft Imaging Solutions, Lakewood, CO, USA). The morphology of mitochondria in granulosa cells was examined.

### 4.6. Immunofluorescence

For mitochondrial localization, granulosa cells were collected and cultured overnight in μ-Slide 8 well plates (ibidi GmbH, Martinsried, Germany) to allow cell attachment. Granulosa cells were stained with 200 nM MitoTracker Green (Molecular Probes, Eugene, OR, USA) for 45 min at 37 °C and cells were then fixed with 4% paraformaldehyde for 20 min and treated with 0.5% Triton X-100 for 20 min, the liquid removed and blocked by 0.5% BSA in PBS 1 h. After empty the well, granulosa cells were counterstained with 4',6'-diamidino-2-phenylindole (DAPI; Sigma, St. Louis, MO, USA) for 10 min at 37 °C, samples were observed by *Delta Vision Elite* Microscope (Applied Precision, GE Healthcare, Olive Branch, MS, USA).

### 4.7. Determination of Mitochondrial DNA (mtDNA) Content in Granulosa Cells

Total DNA was extracted from granulosa cells using a DNeasy kit (Qiagen). mtDNA content was calculated using quantitative real-time PCR as described previously [57,58] by measuring the threshold cycle ratio (Δ*C*_t_) of a mitochondria-encoded gene cytochrome c oxidase subunit 2 (COX2) (L7447, 5'-AATAGAACTTCCAATCCGTA-3', H7726, 5'-AAGGTTAACGCTCTTAGCTT-3') *vs.* a nuclear-encoded gene (18s-rRNA-f, 5'-CGGCTACCACATCCAAGGAA-3', 18s-rRNA-r, 5'-GCTGGAATTACCGCGGCT-3'). Data were expressed as mtDNA/nuclear DNA. All measurements were performed in triplicate.

### 4.8. Metabolite Analytic Assays

Granulosa cells were measured for ATP content by ATPlite™ Luminescence ATP Detection Assay System (Perkin Elmer Life and Analytical Sciences B.V., Groningen, The Netherlands). Granulosa cells were homogenized in 50 µL mammalian cell lysis buffer and then 50 µL substrates solution were added. Adapt the plate to darkness for 10 min and then measure the luminescence. Reactions were normalized to total protein, and metabolite contents were expressed as µM/mg protein.

### 4.9. Detection of Mitochondrial Membrane Potential by Flow Cytometry

Granulosa cells were collected from 4-week-old female mice (*AR^+/+^* and *AR*^−/−^) injected with 7.5 IU PMSG 48 h later. To get positive control from cells treated with CCCP (carbonyl cyanide 3-chlorophenylhydrazone, Sigma Immunochemicals, St. Louis, MO, USA) to depolarized the mitochondrial membrane potential. Cells were incubated with 500 nM JC-1 (5,5',6,6'-tetrachloro-1,1',3,3'-tetraethylbenzimidazolylcarbocyanine iodide, Molecular Probes, Eugene, OR, USA) in the PBS for 15 min at 37 °C in the dark and then analyzed by flow cytometry (BD Biosciences, San Jose, CA, USA). The acquired data were further analyzed using FCS express V4 analysis software.

### 4.10. Assessment of Serum Hormone Levels

To determine the level of estradiol (E2) in the experimental animals, sera were collected from both *AR*^−/−^ and *AR^+/+^* female mice that were previously treated with 7.5 IU PMSG for 48 h and stored at −20 °C before further analysis were carried out. Total E2 level was measured by ELISA kits following the manufacture’s instruction (USCN Life Science, Wuhan, China). The volume of serum used for ELISA was 50 μL, the minimal detectable concentration was 4.38 pg/mL, and the intra- and interassay coefficients of variation were 8.7% and 9.8%, respectively.

### 4.11. Statistical Analysis

Data are presented as mean ± standard error mean (SEM), unless otherwise indicated. Statistical comparisons were made with Student’s *t* test. *p* ≤ 0.05 as considered to be significant.

## Supplementary Materials

Supplementary materials can be found at http://www.mdpi.com/1422-0067/16/05/9831/s1.
