# Supplementary material for: Abnormal Mitochondrial Function and Impaired Granulosa Cell Differentiation in Androgen Receptor Knockout Mice
Source: Int J Mol Sci. 2015 Apr 30;16(5):9831–49. doi: 10.3390/ijms16059831 (PMC4463620; doi:10.3390/ijms16059831)
Supplement: Supplementary file 1 [file ijms-16-09831-s001.pdf]

Supplementary Information

(A)

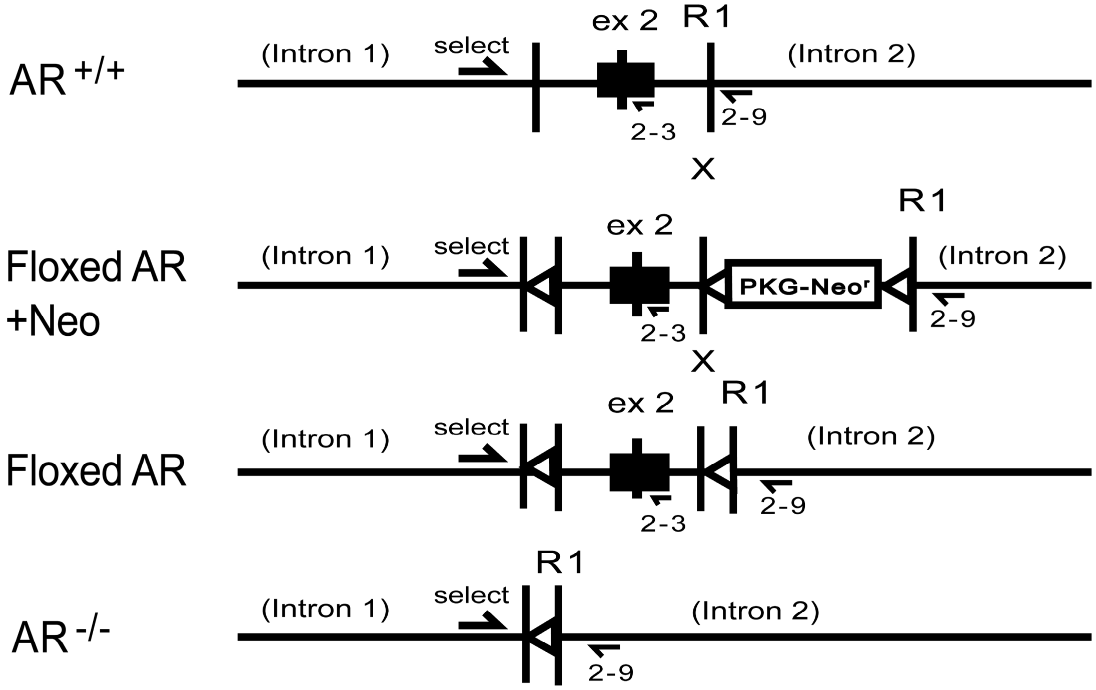

(B)

Genotype ACTB Cre⁺	Primer	PCR product size
-/Y	Select & 2-9	238 bp
-/X	Select & 2-9	238 & 580 bp
X/Y	Select & 2-9	580 bp
X/X	Select & 2-9	580 bp

Figure S1. Genotyping of *AR*$^{-/-}$ mice. We have used the primers "select" and "2–9" to identify *AR*$^{+/+}$ and *AR*$^{-/-}$ female mice in our study. (**A**) Schematic presentation of the DNA construct and primer location in exon 2 area of *AR*$^{+/+}$, *AR*$^{-/-}$, and floxed *AR* genes; (**B**) List of the sizes of PCR product amplified by designed primer pairs.
